# Degradation of betaine aldehyde dehydrogenase transgenic maize BZ-136 straw and its effects on soil nutrients and fungal community

**DOI:** 10.3389/fmicb.2023.1180310

**Published:** 2023-06-06

**Authors:** Xuesheng Liu, Xing Zeng, Yuhang Zhu, Wei Wang, Siqi Huang, Xinxin Qiao, Zhenhua Wang, Hong Di, Juanjuan Qu

**Affiliations:** ^1^College of Resources and Environmental Science, Northeast Agricultural University, Harbin, China; ^2^College of Agronomy, Northeast Agricultural University, Harbin, China

**Keywords:** *BADH* transgenic maize, straw degradation, fungal community, co-occurrence network, FUNGuild

## Abstract

The development of salt-alkali tolerant genetically modified crops represents an important approach to increase grain production in saline-alkali soils. However, there is a paucity of research on the impact of such genetically modified crops on soil microbial diversity. This study aims to investigate the straw degradation of betaine aldehyde dehydrogenase (*BADH*) transgenic maize BZ-136 and its effects on soil chemical properties, fungal community composition, community diversity and ecological function compared to non-transgenic maize Zheng58 straw. The degradation experiments of BZ-136 straw were carried out under a simulated burying condition with saline-alkali soil for 210 days. The results showed that the degradation rate of C and N of BZ-136 straw was significantly faster than that of Zheng58 in the early stage (*p* < 0.05). Compared to Zheng58, the straw degradation of BZ-136 increased the soil available nitrogen (AN), total phosphorus (TP), and available phosphorus (AP) in the early stage (*p* < 0.05). The AN content of soil with BZ-136 straw was 18.16 and 12.86% higher than that of soil with Zheng58 at day 60 and 120 (*p <* 0.05). The TP content of soil with BZ-136 was higher 20.9 and 20.59% than that with Zheng58 at day 30 and 90 (*p <* 0.05). The AP content of soil with BZ-136 was 53.44% higher than that with Zheng58 at day 60 (*p <* 0.05). The straw degradation of BZ-136 increased the OTU number of soil fungal community by 127 (*p <* 0.05) at day 60, and increased Chao1 and Shannon index at day 60 and 180 (*p <* 0.05). The degradation rate of C and N in BZ-136 straw was higher than that in Zheng58 at early stage, which led to the phased increase of soil AN and TP contents, and the obvious changes of relative abundances (RA) of some genera and guilds. These findings are important as they provide insight into the potential benefits of *BADH* transgenic crops in upgrading the soil fertility and the fungal community diversity.

## 1. Introduction

Soil salinization is a widespread problem caused by chemical weathering, salt redistribution and seawater irrigation, etc. Report shows that the area of salinized soil increases at a rate of 1.0–1.5 × 106 hm^2^ per year (Vanhie et al., 2015). Soil salinization constrains the agricultural production by decreasing crop yield and deteriorating crop quality (Liu et al., 2010). Physical or chemical methods are traditionally used to cope with soil salinization, but these methods are cost efficiently and often cause secondary soil salinization. Therefore, scientists try to explore transgenic technology to solve this issue and many salt tolerant crops have been bred by transforming abiotic-stress resistant genes into crop genome.

However, GM (genetically modified) crops can pose multiple environmental risk. Therefore, the risk assessment is essential for the planting of GM crops. The return of transgenic crop straw may release recombinant DNA and its protein to the soil through litter fall (Ceccherini et al., 2003; De Vries et al., 2003), which may be adsorbed onto soil minerals (Crecchio et al., 2005; Cleaves et al., 2011), and ingested by soil microbes (Finkel and Kolter, 2001; Levy-Booth et al., 2008; Alvarez et al., 2010). The research on the impact of GM crops on soil microorganisms is crucial, as these organisms make up over 80% of underground biomass and play a significant role in shaping terrestrial ecosystems (Bruinsma et al., 2003).

However, various biotic and abiotic factors have prevented researchers from reaching a consensus on whether GM crops can affect soil microbes (Guan et al., 2016). On the one hand, some publications have shown that transgenic plants had negative effects on microbial biomass, enzyme activity and microbial diversity (Chen et al., 2012, 2017; Wei et al., 2022). On the other hand, most studies consider these effects are insignificant, and even there is significant, it is temporary.

The betaine aldehyde dehydrogenase (*BADH*) gene encodes a key enzyme for the synthesis of glycine betaine (GB), and its activity will be greatly increased under drought and salt stress, thus causing high amount of GB accumulation in maize (Hanson et al., 1985; Keita et al., 1987), maintaining intracellular osmotic pressure, stabilizing cell membrane structure and finally ensuring maize growth under salt or drought stress (Gorham, 1995).

Except root exudates, the incorporation of *BADH* transgenic maize straw to the field is a main pathway of GB into soil environment, therefore, its potential effects on soil microbes deserve more attention (Xu et al., 2018). The literature on the effects of *BADH* straw on soil microorganisms is particularly sparse (Huang et al., 2020). Both fungal and bacterial communities play important roles in degrading soil xenobiotics (Banerjee et al., 2016; Kumar et al., 2021, 2022). Fungi degrade cellulose and lignin in straw through two extracellular enzyme systems (hydrolases and oxidases) (Sanchez, 2009; Andlar et al., 2018). However, it remains unclear how fungi change with *BADH* transgenic maize degradation. In this study, the effects of straw from *BADH* transgenic maize BZ-136 and non-GMO maize Zheng58 on fungal communities in saline-alkali soil were compared, and straw decomposition and soil nutrient changes were also studied as environmental factors. The results of this study will provide valuable information for evaluating the influence of abiotic-stress resistance transgenic plant straw return to the field on soil ecology.

## 2. Materials and methods

### 2.1. Soil and maize plants

The soil (saline-alkali chernozem) used in this experiment was collected from Lindian of Heilongjiang province, China. The properties of soil were determined as pH 8.08, conductivity 0.653 ds·m^−1^, organic carbon (OC) 2.39%, total nitrogen (TN) 0.60 g·kg^−1^, available nitrogen (AN) 80.92 mg·kg^−1^, total phosphorus (TP) 0.63 g·kg^−1^, available phosphorus (AP) 12.64 mg·kg^−1^, available potassium (AK) 118.73 mg·kg^−1^, and Na^+^ 98.5 mg·kg^−1^ (Page et al., 1982). The soil was divided into two parts, one for maize planting and the other for straw burying. One transgenic maize cultivar BZ-136 (expressing *BADH* gene) and its parental cultivar Zheng58 were planted with above saline-alkali soil in a greenhouse under normal agricultural management (Di et al., 2015). Maize straws were collected after maize plants were mature, and the initial characteristics of BZ-136 and Zheng58 straw were determined as cellulose 39.19 and 38.76%, lignin 17.92 and 18.50%; C/N 52.81 and 57.33; P 6.36 g·kg^−1^ and 5.04 g·kg^−1^; K 14.36 g·kg^−1^ and 15.77 g·kg^−1^; GB 7.63 mg·g^−1^ and 0.86 mg·g^−1^.

### 2.2. Experimental design of straw degradation

The degradation experiment was conducted at Northeast Agricultural University (126°73′ e and 45°75′ n) from November 2017 to May 2018 (210 days). The straws were air-dried at 60°C, cut into 1 cm of segments, and put into nylon bags with soil (3 g of straws thoroughly mixed with 97 g of soil, and totally 21 bags for each kind of maize). Then, the 3 bags containing the same kind of straws were buried into soil bucket to a depth of 15 cm (28 cm in diameter and 32 cm in depth). Soil moisture was sustained at 60–80% of the field water holding capacity, and room temperature was maintained at 25 ± 1°C. For both kinds of maize, bags in the same bucket were taken out at day 30, 60, 90, 120, 150, 180, and 210, respectively. The straw samples were carefully separated from soil and dried to constant weight at 60°C for further chemical property analysis. The soil samples were divided into two parts, one was stored at −80°C for soil DNA extraction and fungal community analysis, the other part was air-dried for chemical property determination.

### 2.3. Chemical properties and elements content of straws

The straws were crushed, passed through a 0.5 mm sieve, weighed and put into a muffle furnace at 500°C, then ash mass was weighed and ash content was calculated (Lu et al., 2010). The C content was determined by total organic carbon analyzer (TOCA) (TOC-L, Tsushima, Japan). The N, P and K contents were measured by Kjeldahl™ 8,400 Auto Sample Systems (FOSS Tecator AB, Sweden), flame photometer (6400A, Rainbow, China) and Molydenum-antimony-D-iso-ascorbie-acid-colorimetry respectively, after straws were digested in concentrated H_2_SO_4_ (Varley, 1966).

### 2.4. Chemical properties of soil

The OC content was determined according to Walkley and Black’s method based on the reaction with K_2_Cr_2_O_7_ and H_2_SO_4_ (Walkley and Black, 1934). The AK content was directly measured with a flame photometer after the soil was extracted with neutral 1 M NH_4_OAc (Lacy, 1965). TN and AN were determined according to Kjeldahl method (Bremner and Mulvaney, 1982) and alkali hydrolysis-diffusion method (Cornfield, 1960), respectively. TP and AP were determined by the molybdenum antimony colorimetric method after the soil was digested with HClO_4_-H_2_SO_4_ and treated with 0.5 M Na_2_HCO_3_.

### 2.5. Fungal high-throughput sequencing and functional prediction

The soil DNA was extracted with TIANamp Soil DNA Kit, according to the manufacturer’s instructions. The primers ITS1-F (5′-CTTGGTCATTTAGAGGAAGTAA-3′) and ITS2-R (5′-GCT GCTGCGTTCTTCATCGATGC-3′) were used to amplify the ITS1 regions of the fungal ITS rRNA genes. The DNA library was constructed with PrimeSTAR® GXL DNA Polymerase (TaKaRa) according to the manufacturer’s protocols. Library was quantified using NGS™ dsDNA HS Assay Kit by Qubit® 3.0 Fluorometer (Life Technologies, USA). The qualified libraries were sequenced on Illumina Miseq platform by 250-bp mode. Inhouse Perl scripts were used to analyze alpha diversity. Graphical representation of the relative abundance of fungal diversity from phylum to species can be visualized using Krona chart. Using CoNet plug-in of Cytoscape to draw network graph and analyze the correlation between environmental factors and relative abundance of OTUs. OTUs information was uploaded to the FUNGuild database for functional groups (guild) prediction.

### 2.6. Statistical analysis

Data were expressed as mean ± standard deviation. SPSS 23.0 was used for Pearson coefficient and other data analysis. One-way ANOVA was used to compare the data between groups, and *p* < 0.05 presents significant difference. ANOSIM differences were calculated using the vegan package in R software. Cytoscape 3.8.0 was used to infer correlation between environmental factors and relative abundance of OTUs.

### 2.7. Accession numbers

Structure factors have been deposited in the Protein Data Bank with accession number PRJNA563165.

## 3. Results

### 3.1. Changes in ash-free dry mass and element content of straws

**Figure 1:**
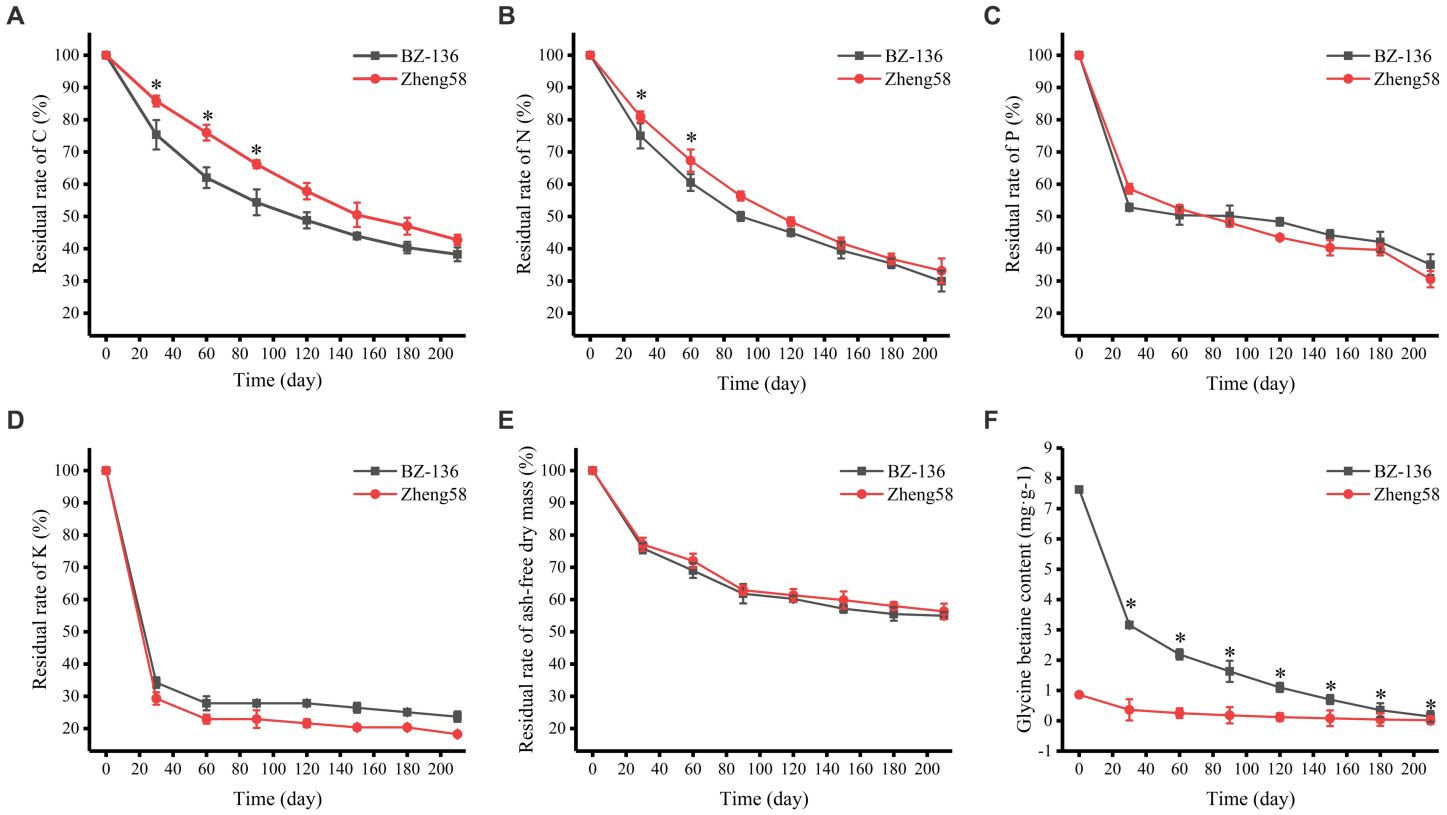
Changes in residual rate of two straws components over time. **A**, residual rate of C; **B**, residual rate of N; **C**, residual rate of P; **D**, residual rate of K; **E**, residual rate of ash-free dry mass; **F**, glycine betaine content. * represents significant difference (*P* < 0.05).

According to the degradation rate of straws shown in Figure 1, the degradation process may be divided into two stages: the early stage is a rapid degradation period from day 1 to 30, during which ash-free dry mass (AFDM) and the contents of C, N, P, and K decreased rapidly; the later stage is a slow degradation period from day 30 to 210, during which the degradation rate decreased significantly. The contents of C, N, P, and K in straws decreased gradually with time, among which K content decreased fastest, followed by P, N and C. During the early stage, C, N, and P content of BZ-136 decreased more markedly than that of Zheng58 by 10.44, 6.14, 5.74%, respectively, at day 30. It was noteworthy that only K content of Zheng58 decreased more than that of BZ-136 by 4.94% in the first stage. During the later stage, C content of BZ-136 was 13.96 and 11.84% lower than Zheng58 (*p* < 0.05), N content of BZ-136 was 6.81 and 6.33% lower than Zheng58 (*p* < 0.05) at day 60 and 90, respectively. Apparently, P and K contents of BZ-136 were generally higher than those of Zheng58 in most period of the later stage, but there was no significant difference between two straws. In brief, the changes of all element contents between different straws gradually reduced over time, and the differences of C and N content of two straws were narrowed to 4.50 and 3.23%, respectively (*p* > 0.05), leading to a similar AFDM of different straws in the whole period. The content of GB of Zheng58 sustained at a very low level, while that of BZ-136 continuously declined to 0.14 mg·g^−1^ at the end of degradation.

### 3.2. Soil chemical properties

**Figure 2:**
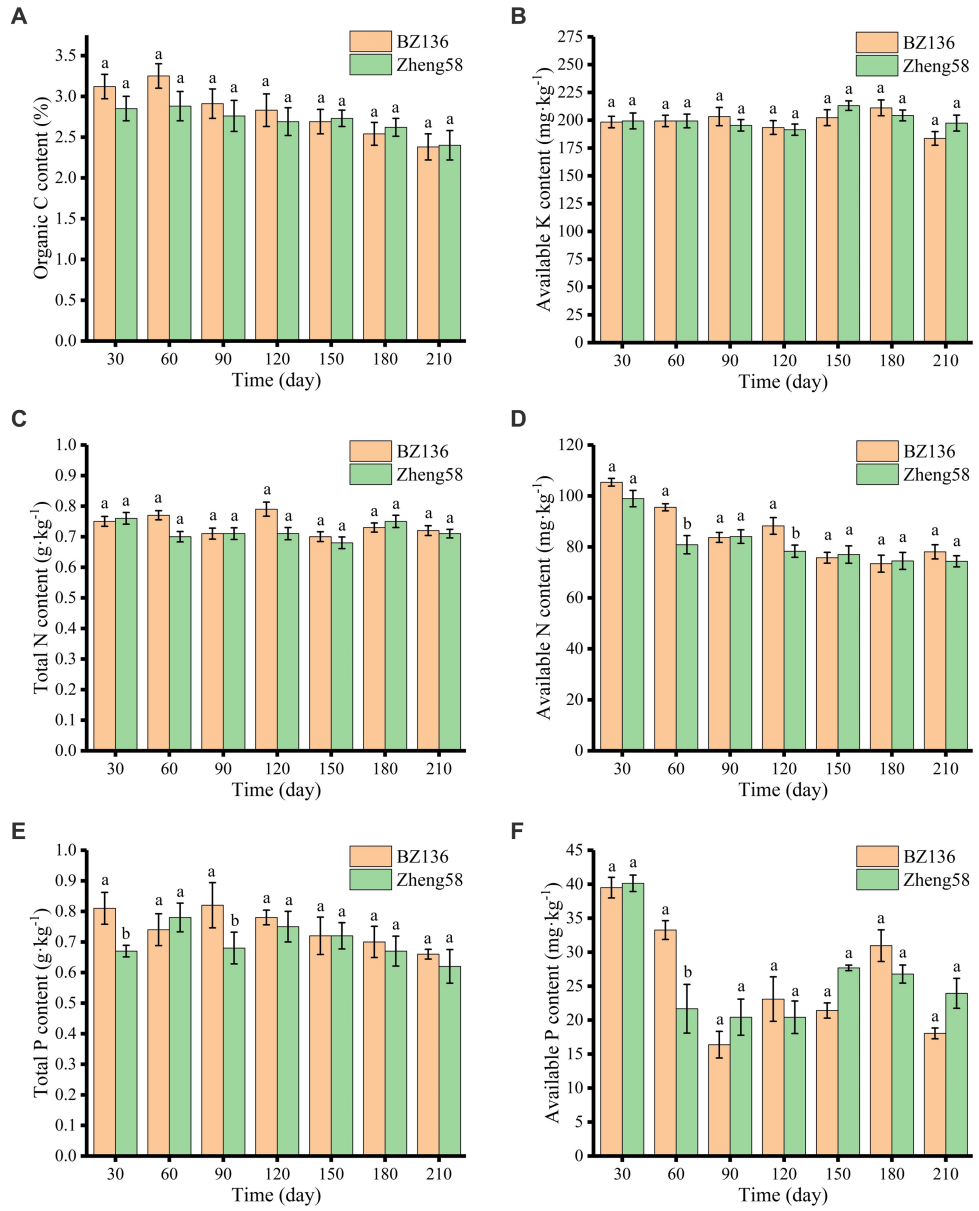
Changes of soil OC **(A)**, AK **(B)**, TN **(C)**, AN **(D)**, TP **(E),** and AP **(F)** content over time. The different letters represent significant differences (*P* < 0.05).

As shown in Figure 2A, OC content of different soil samples decreased slowly over time, and there was no significant difference in OC content between different soil samples. In most of time, OC content of soil with BZ-136 straw was slightly higher than that with Zheng58 by 0.27, 0.37, 0.15, and 0.14% at day 30, 60, 90, and 120 (*p* > 0.05), respectively. It was that OC content of soil with BZ-136 was 0.01% lower than its initial value, and this scenario did not appear in soil with Zheng58.

As shown in Figure 2B, the AK contents of soil with BZ-136 and Zheng58 straw were 183.62 mg·kg^−1^ to 211.11 mg·kg^−1^ and 191.47 mg·kg^−1^ to 213.08 mg·kg^−1^, respectively. and there were no significant differences between soil samples and degradation time.

As shown in Figures 2C,D, the change in soil TN content of both soil samples was unremarkable. Compared with Zheng58, the degradation of BZ-136 straw did not cause obvious change in TN content, but had a greater impact on AN. The AN content of soil with BZ-136 straw was 18.16 and 12.86% higher than that of soil with Zheng58 at day 60 and 120 (*p* < 0.05).

As shown in Figures 2E,F, the change in TP content of soil with BZ-136 showed a down-up-down trend, specifically, TP content was 0.81 g·kg^−1^ at day 30 and 0.82 g·kg^−1^ at day 90, which was 0.07 g·kg^−1^ and 0.08 g·kg^−1^ higher than that at day 60, respectively (*p* < 0.05). The TP content of soil with BZ-136 was higher 20.9 and 20.59% than that with Zheng58 at day 30 and 90 (*p* < 0.05), but there was no significant difference between them after 120 days. The AP contents of both soils fluctuated at different sampling days, AP content of soil with BZ-136 was 53.44% higher than that with Zheng58 at day 60 (*p* < 0.05), but difference between two soil samples disappeared after day 90.

### 3.3. Soil fungal community structure and function

#### 3.3.1. Diversity and richness of fungal community

**Figure 3:**
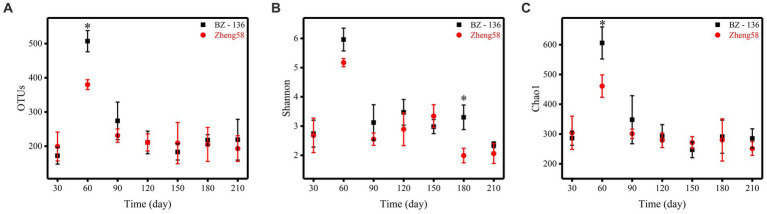
Alpha diversity statistics. Comparison of OTU numbers, Shannon index, and Chao1 index between the two components of straws, represented by **A**, **B**, and **C**, respectively. * represents significant difference (*P* < 0.05).

A total of 4,743,109 effective sequences in 42 soil samples was retrieved from the optimized database, and the average length of each sample was 274.31. Based on the similarity level of 97%, all sequences were divided into OTUs for biological information analysis. The number of OTUs, Shannon diversity index and Chao1 richness index obtained from the optimized sequence database were shown in Figure 3. The number of OTUs in the soil with BZ-136 straw was 507 at day 60, while that with Zheng58 was 380 (*p* < 0.05), Shannon index of the former was 3.30 at day 180, which was 1.31 higher than that of the latter (*p* < 0.05), and Chao1 index of the former at day 60 was 606, 145 more than that of the latter (*p* < 0.05). The highest values of OTUs, Shannon and Chao1 of different soils all appeared at day 60.

#### 3.3.2. Fungal community composition at phylum level

**Figure 4:**
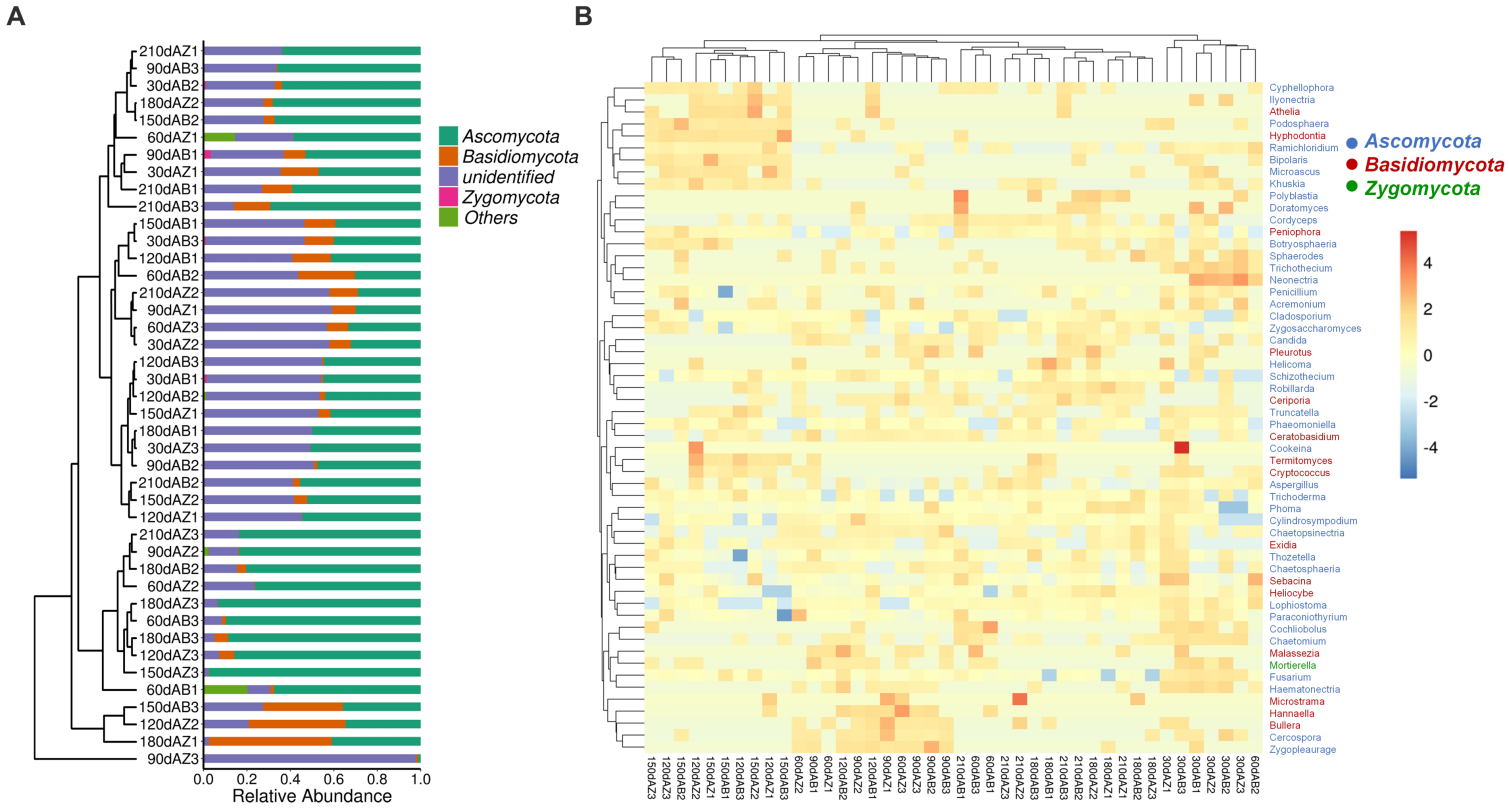
**(A)** Fungal community composition of different soil at phylum level, 30 dAB represents soil with BZ-136 straw at day 30, 30 dAZ represents soil with Zheng58 straw at day 30, and so on; **(B)** Heatmap of fungal community composition at genus level of different soils.

The phyla listed in Figure 4A were top 4 phyla with high relative abundances (RA). Except for unidentified phyla which cannot be compared with sequence in the database under the confidence threshold, the phyla were Ascomycota, Basidiomycota and Zygomycota in order of RA. During the whole degradation period, there was no significant difference of fungal community composition at phylum in different soil samples.

#### 3.3.3. Fungal community composition at genus level

As shown in Figure 4B, 56 dominant genera were identified in the heatmap, and their RA was represented by the color intensity as illustrated in the figure. The results showed that the fungal community composition of soil with BZ-136 straw had lower similarity with soil with Zheng58 at day 60. In detail, the RA of *Cyphellophora* (only appeared in soil with BZ-136) was 0.0016%, that of *Bullera* (only appeared in soil with Zheng58) was 0.0020% (*p* < 0.05) at day 60, that of *Mortierella* (only appeared in soil with BZ-136) was 0.63% (*p* < 0.05) at day 30; that of *Truncatella* and *Sebacina* of the former was 0.00092 and 0.068%, respectively, which was 0.00092 and 0.057% higher than that of the latter (*p* < 0.05) at day 90; and at day 150 that of *Acremonium* of the former was 0.56% higher than that of the latter (*p* < 0.05). Except at day 90, the RA of *Penicillium* and *Fusarium* of the former was higher than that of the latter at most degradation stages (*p* > 0.05), while that of *Aspergillus* of the former was higher than that of the latter in the early stage of degradation and lower in the later stage (*p* > 0.05). It is worth mentioning that *Zygosaccharomyces* as a salt and osmotic tolerant genus showed a high relative abundance in different soils.

#### 3.3.4. The environmental factors influencing OTU relative abundance

**Figure 5:**
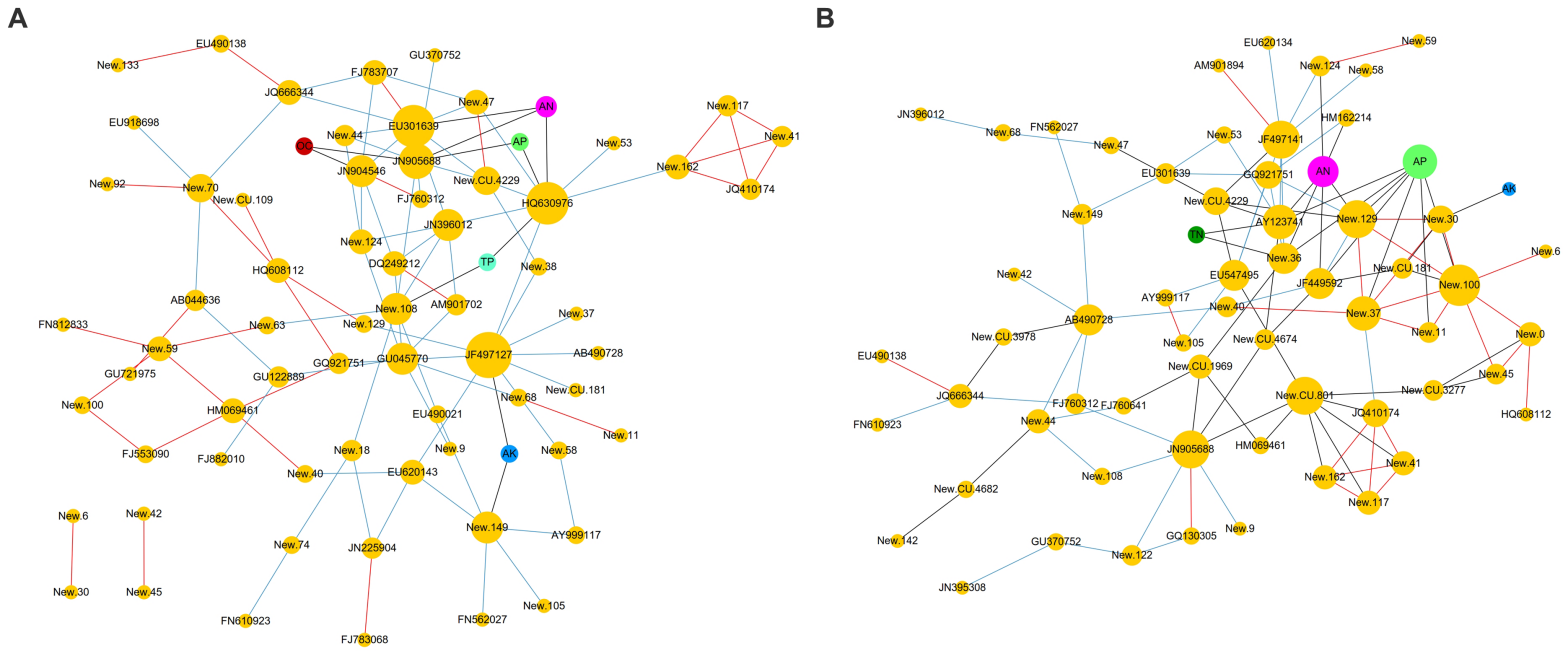
Co-occurrence network analysis between OTUs and environmental factors **(A)** BZ-136 group; **(B)** Zheng58 group.

The co-occurrence network analysis (Figure 5) clearly revealed the correlations between the RA of fungal OTUs and environmental factors. In BZ-136 group (Figure 5A), the correlation between TN and OTUs was not shown in the figure, because their coefficient value was lower than 0.3. Other factors, such as OC, AK, TN, AN, TP, and AP, had the strongest correlation with New.ReferenceOTU63(0.407), New.ReferenceOTU63(0.533), JN225904(0.289), JN905688(0.635), New.ReferenceOTU108(0.382), and JN905688(0.553), respectively. AN had the greatest effect on OTUs, followed by AP. In Zheng58 group (Figure 5B), the correlations between OC and TP with OTUs were not shown in the figure, because their coefficients were all lower than 0.3. OC, AK, TN, AN, TP, and AP had the strongest correlation with FJ882010(0.406), New.ReferenceOTU30(0.339), New.CleanUp.ReferenceOTU4674(0.360), New.ReferenceOTU129(0.674), FJ882010(0.355) and New.CleanUp.ReferenceOTU181(0.745), respectively. Obviously, according to the coefficient values, AP had the greatest effect on OTUs, followed by AN.

In BZ-136 group, JN905688, New.ReferenceOTU122, New.CleanUp.ReferenceOTU109 and HQ630976 were the 4 OTUs with the highest correlation value with AN and AP. In Zheng58 group, 11 OTUs such as New.ReferenceOTU129, FN812833, New.ReferenceOTU122, New.CleanUp.ReferenceOTU181, New.ReferenceOTU36 were more related to the above 2 environmental factors. The detailed correlation coefficient between RA of OTUs and environmental factors in BZ-136 and Zheng58 group was listed in Supplementary Table S1.

Each orange node represents an OTU. The prefix New. CleanUp. ReferenceOTU of OTU was abbreviated as New. CU., and New. ReferenceOTU was abbreviated as New. OTU. Other color nodes represent environmental factors, OC: Organic carbon; AK: Available potassium; TN: Total nitrogen; AN: Available nitrogen; TP: Total phosphorus; AP: Available phosphorus. The correlation between OTUs was represented by red edges (positive) and blue edges (negative). Black edges indicated that the correlation values between OTU and environmental factors were greater than 0.3.

#### 3.3.5. Probe of fungal functional group

**Figure 6:**
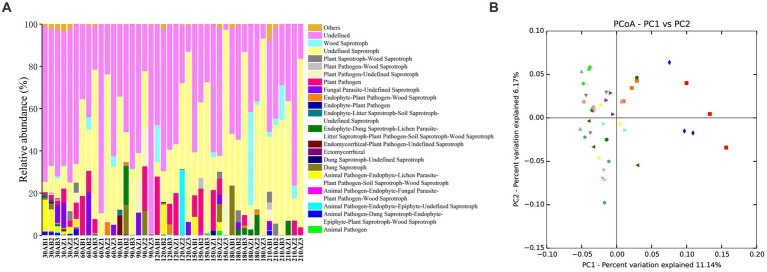
**(A)** Functional groups (Guilds) of fungal OTUs by FUNGuild database; **(B)** principal co-ordinates analysis (PCoA) of fungal community composition in different soils.

In this study, FUNGuild was used to classify soil fungi OTUs, and 3 trophic modes and 65 guilds were obtained. Excluding the lower abundant guilds, there were still 20 guilds in Figure 6A. The RA of Endophyte-Litter Saprotroph-Soil Saprotroph-Undefined Saprotroph of soil with BZ-136 straw was higher than that with Zheng58 from day 30 to 120, especially 0.63% higher at day 30 (*p* < 0.05) and 11.68% at day 60 (*p* < 0.05), but at the end of degradation, it became lower than that of the latter (*p* > 0.05). The RA of Ectomycorrhizal fungi of soil with BZ-136 straw was lower than that with Zheng58 straw at day 30, 90, 120, 150 and 180, but there was a significant difference between them at day 120 (*p* < 0.05). In addition, due to the importance of saprophytic fungi in the decomposition process of plant litter, it was not surprising that the RA of Undefined Saprotroph was highest in all samples, and the RA of Undefined Saprotroph of both soils has a trend of up-down-up over time. In the soil with BZ-136 straw, the peak value (45%) of the RA of Undefined Saprotroph appeared at day 60 and 180, while in the soil with Zheng58 straw, the peak value (48%) of the RA of Undefined Saprotroph appeared at day 120, 180, and 210. There was no significant difference in the RA of Undefined Saprotroph between different soils at the same period. A total of 419 OTUs were assigned to Undefined Saprotroph guild, of which 390 belong to some genera of Ascomycota phylum (e.g., *Aspergillus*, *Penicillium*, *Zygosaccharomyces*, *Trichoderma*, *Khuskia,* and *Thozetella*). There was no significant difference in RA of Wood Saprotroph between different soils at the same period. Except for day 150 and 180, the RA of the guild in soil with BZ-136 straw was higher than that with Zheng58 (*p* > 0.05). A total of 51 OTUs were assigned to Wood Saprotroph guild, of which 42 belonged to some genera of Basidiomycota phylum (e.g., *Ceriporia*, *Schizophyllum,* and *Heliocybeceriporia*), and 9 OTUs belonged to Ascomycota phylum.

#### 3.3.6. Community composition similarity

Principal co-ordinates analysis (PcoA) was used to analyze the similarity and difference of community composition. As shown in Figure 6B, symbols of the same color and shape represent 3 repetitions of the same soil sample. According to ANOSIM analysis, both BZ-136 and Zheng 58 straw degradation did not cause significant changes in soil fungal community composition.

## 4. Discussion

To cope with the adverse impact of soil salinization on plant growth, scientists developed novel salt-tolerant crops by transgenic technology (Di et al., 2015) and evaluated the risk of these transgenic plants on soil environment and microbial activities (Bai et al., 2019). The study conducted by Sahoo and Tuteja (2013) demonstrated that no discernible trends were observed in soil properties (pH, EC, organic C, and N) at any growth stage of two lines of maize containing the BADH gene, either in neutral or saline-alkaline soil. The study by Zeng et al. (2022) demonstrated that the cultivation of BADH genetically modified maize had no effect on soil enzyme activity and nematode communities.

However, few studies have addressed the potential influence of the residue degradation of these transgenic crops on microbial community structure and diversity. The *BADH* gene encodes a key biosynthesis enzyme of a quaternary ammonium compound GB that can increase the intracellular osmotic pressure in response to different stresses in plants (Mao et al., 2019). *BADH* transgenic plants not only possess higher tolerance to abiotic stresses, but also have a higher N content in their straws, which directly determines degradation rate of plant residue. In this study, the content of GB of transgenic maize straw was significantly higher than that of non-transgenic straw (*p* < 0.05). Many fungi, such as *Aspergillus fumigatus* (Lambou et al., 2013), *Penicillium fillutanu* (Park et al., 1999) and *Aspergillus nidulans* (Kacprzak et al., 2004), can use GB as carbon and nitrogen sources (Wargo, 2013), some even ingest environmental GB instead of synthesis in person (Galinskie and Truper, 1982). Since straw degradation is dominated by microbes, the degradation rates of C and N of BZ-136 straw were significantly higher than those of Zheng58 in the early stage (*p* < 0.05), due to higher GB content in transgenic maize. Pearson analysis also showed that there was a significant correlation between GB content and the changes of C and N in the two kinds of straws (*p* < 0.01), especially in the transgenic straw, the correlation coefficient between them was as high as 0.941 (*p* = 0.002) and 0.922 (*p* = 0.003), respectively. Moreover, carboxyl of GB can precipitate Ca^2+^ and Mg^2+^ in soil, thus reduce the osmotic pressure of saline-alkali soil and create a suitable growth condition for microbes. Among tested 4 elements, the content of K decreased fastest, a plausible explanation is that about 80% of K absorbed by crops is stored in straw, which will be released into the soil quickly with the straw degradation (Saha et al., 2010).

The degradation of *BADH* transgenic maize straw with higher nitrogen content might inhibit the mineralization of soil organic carbon. As displayed in Figure 2, the content of OC in soil with transgenic straw was slightly higher than that with non-transgenic (*p* > 0.05) in the early stage, while it greatly reduced in the later stage probably due to the enhanced protection of clay particles to OC (Six et al., 2002), which led to a decrease in straw degradation rate. At day 60, the AN content of soil with transgenic straw was significantly higher than that of soil with non-transgenic straw (*p* < 0.05). This is because nitrogen input can also enhance soil nitrification, thus increasing NO_3_^−^-N and NH_4_^+^-N, and reducing soil pH (Turner and Henry, 2009; Hogberg et al., 2010), which plays an indirect role in improving salinealkali soil. Pearson correlation coefficient also showed that there was a high correlation between GB content in straw and AN content in soil (*r* = 0.893, *p* < 0.01). Of course, if the input of exogenous nitrogen exceeded the demand of the ecosystem, the degradation of lignin by white rot fungus will be inhibited, resulting in the transformation of microbial community composition to low efficiency, which was unfavorable for the degradation of crop residues (Prescott and Blevins, 2004). In this study, it was apparent to find that the degradation of *BADH* transgenic straws increased soil TP and AP content in most period of degradation (*p* < 0.05). Firstly, AMF colonized at roots of *BADH* transgenic maize enhanced P uptake by crops and correspondingly increased P content in straws (Kim and Weber, 1985; Bolan et al., 1987). The second reason is that the input of exogenous nitrogen might promote phosphatase activity (Johnson et al., 1998; Henry et al., 2005; Stursova et al., 2006), and subsequently facilitated the transformation of organic phosphorus into inorganic phosphorus as well as unavailable to available phosphorus. However, the degree of this influence was periodic and was recovered in the later stage.

The co-occurrence network analysis showed that compared with other soil chemical properties, AN and AP content had a greater impact on the RA of soil fungal OTUs. In addition, in the early stage, soluble sugars were released into soil with straw residue degradation. Bacteria, especially those of *r*-strategy, used these soluble sugars for rapid proliferation, and released antibiotics and chitinase that inhibited the fungi growth. Therefore, the highest fungal OTUs number appeared at day 60 rather than day 30. After the easy-to-use substances were decomposed, cellulose and lignin were the main energy substances of microbes. At this time, fungi, especially those of K-strategy, used them for self-proliferation, and their number increased gradually (Meidute et al., 2008; Mcguire and Treseder, 2010; Marschner et al., 2011). After 60 days, the differences of OTUs, Shannon and Chao1 in different soil almost disappeared, which indicated that the effect of *BADH* maize straw degradation on the alpha diversity of soil fungal community was temporary. Ascomycota was the most abundant phylum in this experiment. The trophic modes of its members are saprophytism, parasitism and symbrosis. Saprophytic Ascomycota can cause mildew of wood, food, cloth and leather, and decomposition of animal and plant residues. However, the degradation of *BADH* transgenic maize straw had no significant effect on the soil fungal community composition at phylum level, and had little effect at the genus level as well. This study only found that the RA of 6 genera (*Cyphellophora*, *Truncatella, Sebacina*, *Mortierella*, *Acremonium* and *Bullera*) were different among different soils (*p* < 0.05). Some species of *Sebacina* and *Acremonium* have the function of cellulose degradation (Kohler et al., 2015), and their RA at day 90 and 150 in the soil with transgenic straw were higher than that with non-transgenic (*p* < 0.05). The other 4 genera were not found to be related to the straw degradation, which needs further study. Due to the effect of GB, the *BADH* transgenic maize had more vigorous growth in saline-alkali soil than non-transgenic, as well as a better developed root system, which was more favorable for the symbiosis of endophytic fungi, resulting in more nutrients absorption especially phosphorus. In addition, the degradation rate of transgenic maize straw was faster than that of non-TRANSGENIC in the early stage, resulting in the RA of many saprophytic fungi increased. Therefore, compared with the non-transgenic, the addition of transgenic straw increased the RA of Endophyte-Litter Saprotroph-Soil Saprotroph-Undefined Saprotroph guild at day 30, which is noteworthy that fungi in this guild have strong cellulose decomposing ability, such as *Aspergillus*, *Penicillium,* and *Trichoderma*. Generally, in the initial stage, the carbohydrates and proteins in straws are degraded preferentially, while cellulose and other substances are difficult to be used. Therefore, the RA of Undefined Saprotroph was the lowest at day 30, and exhibited a rising-falling-rising trend in the whole process. The results of Kimura and Tun (1999) showed that there were complex microbial community changes in the process of straw degradation, which needs further study. It is worth noting that the decrease of microbial diversity does not necessarily affect the normal function of soil (Wertz et al., 2007), and most of the microbes exist functional redundancy, that is, the ecological functions of some species overlap to a certain extent. When a species is removed, the diversity is reduced, the ecological function of the soil will remain unchanged or close to the normal state (Wohl et al., 2004).

This study only examined the short-term changes in soil environment and fungal community. Future research could consider long-term observations of the effects of transgenic straw on soil microbial community, in order to better understand the mechanisms by which different straw types influence soil microbial community structure and function. Additionally, the results of this study indicated that the decomposition of transgenic maize straw affected the release rate of certain nutrients in the soil. Future research could investigate whether this effect is caused by microbial decomposition or other mechanisms, such as chemical reactions or physical processes.

## 5. Conclusion

The degradation rate of C and N in BZ-136 straw was significantly faster than that of Zheng58 in the early stage, but there was little difference in P, K, and AFDM. In the early stage, the degradation of BZ-136 straw significantly increased the content of AN, TP, and AP in soil. There was no significant difference in fungal community composition at phylum level in soils with different straws. At genus level, compared Zheng58 straw, the degradation of BZ-136 straw increased the RA of *Mortierella* at day 30; increased that of *Cyphellophora* and decreased that of *Bullera* at day 60; increased that of *Truncatella* and *Sebacina* at day 90. The co-occurrence network analysis of combined environmental factors showed that the RA of soil fungal OTUs with different straws had stronger correlation with soil AN and AP contents. The RA of fungal Endophyte-Litter Saprotroph-Soil Saprotroph-Undefined Saprotroph guild in soil with BZ-136 straw increased significantly at day 30, and that of Plant Pathogen guild increased significantly at day 60, and that of Ectomycorrhizal guild reduced significantly at day 120, but all these changes were temporary.

In summary, this study investigated the impact of different types of straw on soil fungal community composition and nutrient release. The results showed that the degradation rate of carbon and nitrogen in BZ-136 straw was faster than that of Zheng58 in the early stage, and the degradation of BZ-136 straw significantly increased the content of some soil nutrients. At the genus level, the degradation of BZ-136 straw had a significant impact on the relative abundance of several fungal genera. However, there was no significant difference in fungal community composition at the phylum level between the soils with different straws. The co-occurrence network analysis revealed that the fungal OTUs with different straws had a stronger correlation with soil nutrient contents. Furthermore, the further research could explore the long-term effects of genetically modified straw on soil microbial communities and the mechanisms underlying the effects of straw decomposition on nutrient release.

## Data availability statement

The datasets presented in this study can be found in online repositories. The names of the repository/repositories and accession number(s) can be found in the article/Supplementary material.

## Author contributions

JQ was responsible for conceptualization including ideas, formulation of overarching research goals and aims. XL and XZ were responsible for preparation, creation and presentation of the published work, specifically writing the initial and revised manuscript. YZ, WW, SH, and XQ were responsible for the preparation of reagents, instrumentation, computing resources and other analysis tools. HD has participated in the data analysis and applied statistical and mathematical techniques to analyze the research data. ZW was responsible for supervision of the research activity planning and execution. All authors contributed to the article and approved the submitted version.

## Funding

This work was supported by the Major Genetically Modified Organism Breeding Project of China (No. 2016ZX08011-003), China Postdoctoral Science Foundation (2021M700742), and Heilongjiang Postdoctoral Foundation (LBH-Z21109).

## Conflict of interest

The authors declare that the research was conducted in the absence of any commercial or financial relationships that could be construed as a potential conflict of interest.

## Publisher’s note

All claims expressed in this article are solely those of the authors and do not necessarily represent those of their affiliated organizations, or those of the publisher, the editors and the reviewers. Any product that may be evaluated in this article, or claim that may be made by its manufacturer, is not guaranteed or endorsed by the publisher.

## Abbreviations

BADH, Betaine aldehyde dehydrogenase
AFDM, Ash-free dry mass
AK, Available potassium
TN, Total nitrogen
OC, Organic carbon
AN, Available nitrogen
TP, Total phosphorus
AP, Available phosphorus
RA, Relative abundances
GB, Glycine betaine
TOCA, Total organic carbon analyser
PCoA, Principal co-ordinates analysis
