# Supplementary material for: Degradation of betaine aldehyde dehydrogenase transgenic maize BZ-136 straw and its effects on soil nutrients and fungal community
Source: Front Microbiol. 2023 Jun 6;14:1180310. doi: 10.3389/fmicb.2023.1180310 (PMC10279975; doi:10.3389/fmicb.2023.1180310)
Supplement: Supplementary file 1 [file Table_1.DOCX]

Tab. S1 Correlation coefficient between RA of OTUs and environmental factors in co-occurrence network analysis, (a) BZ-136 group, (b) Zheng58 group. OC: Organic carbon; AK: Available potassium; TN: Total nitrogen; AN: Available nitrogen; TP: Total phosphorus; AP: Available phosphorus.

(a)

| BZ-136 Group | OC | AK | TN | AN | TP | AP |
| --- | --- | --- | --- | --- | --- | --- |
| New.ReferenceOTU41 | 0.109 | 0.000 | 0.040 | 0.162 | 0.096 | 0.020 |
| EU489956 | 0.005 | 0.021 | 0.000 | 0.050 | 0.004 | 0.063 |
| EU490138 | 0.149 | 0.041 | 0.145 | 0.149 | 0.073 | 0.136 |
| New.ReferenceOTU0 | 0.012 | 0.016 | 0.284 | 0.029 | 0.000 | 0.011 |
| HQ832844 | 0.004 | 0.026 | 0.001 | 0.052 | 0.000 | 0.005 |
| New.ReferenceOTU159 | 0.002 | 0.000 | 0.000 | 0.017 | 0.000 | 0.097 |
| New.ReferenceOTU30 | 0.062 | 0.050 | 0.000 | 0.065 | 0.001 | 0.014 |
| FN610923 | 0.058 | 0.019 | 0.129 | 0.008 | 0.042 | 0.016 |
| EU620134 | 0.001 | 0.002 | 0.003 | 0.006 | 0.006 | 0.033 |
| New.ReferenceOTU47 | 0.021 | 0.187 | 0.017 | 0.221 | 0.008 | 0.054 |
| FN812833 | 0.014 | 0.000 | 0.042 | 0.010 | 0.002 | 0.002 |
| New.ReferenceOTU59 | 0.138 | 0.001 | 0.006 | 0.092 | 0.096 | 0.000 |
| New.ReferenceOTU45 | 0.052 | 0.020 | 0.088 | 0.020 | 0.016 | 0.022 |
| HM069461 | 0.002 | 0.023 | 0.000 | 0.032 | 0.051 | 0.102 |
| New.ReferenceOTU142 | 0.004 | 0.012 | 0.013 | 0.005 | 0.017 | 0.008 |
| AY999117 | 0.009 | 0.207 | 0.008 | 0.022 | 0.001 | 0.027 |
| FJ783707 | 0.081 | 0.002 | 0.016 | 0.211 | 0.095 | 0.199 |
| New.ReferenceOTU100 | 0.003 | 0.042 | 0.077 | 0.000 | 0.000 | 0.029 |
| AM901894 | 0.009 | 0.098 | 0.091 | 0.002 | 0.001 | 0.053 |
| EU301639 | 0.238 | 0.008 | 0.009 | 0.381 | 0.009 | 0.294 |
| FJ783068 | 0.005 | 0.002 | 0.155 | 0.001 | 0.059 | 0.097 |
| New.ReferenceOTU133 | 0.001 | 0.102 | 0.001 | 0.067 | 0.002 | 0.008 |
| AY123741 | 0.001 | 0.068 | 0.008 | 0.009 | 0.010 | 0.014 |
| New.ReferenceOTU79 | 0.020 | 0.035 | 0.261 | 0.013 | 0.017 | 0.000 |
| HM162214 | 0.160 | 0.109 | 0.135 | 0.082 | 0.060 | 0.001 |
| JN904546 | 0.397 | 0.044 | 0.054 | 0.134 | 0.299 | 0.006 |
| New.ReferenceOTU105 | 0.025 | 0.242 | 0.003 | 0.081 | 0.002 | 0.007 |
| New.ReferenceOTU108 | 0.058 | 0.087 | 0.000 | 0.000 | 0.382 | 0.053 |
| HQ630976 | 0.263 | 0.087 | 0.069 | 0.416 | 0.349 | 0.325 |
| New.ReferenceOTU18 | 0.078 | 0.015 | 0.180 | 0.005 | 0.043 | 0.009 |
| FJ466722 | 0.044 | 0.000 | 0.176 | 0.036 | 0.000 | 0.003 |
| GU045770 | 0.045 | 0.164 | 0.000 | 0.041 | 0.084 | 0.075 |
| AM901702 | 0.005 | 0.213 | 0.000 | 0.020 | 0.294 | 0.141 |
| JF497127 | 0.000 | 0.347 | 0.005 | 0.025 | 0.006 | 0.021 |
| JF439466 | 0.000 | 0.004 | 0.086 | 0.000 | 0.002 | 0.002 |
| New.ReferenceOTU11 | 0.001 | 0.204 | 0.003 | 0.012 | 0.000 | 0.015 |
| AB354285 | 0.048 | 0.032 | 0.002 | 0.006 | 0.083 | 0.065 |
| EU918698 | 0.018 | 0.042 | 0.198 | 0.039 | 0.099 | 0.032 |
| EU490021 | 0.070 | 0.002 | 0.007 | 0.186 | 0.072 | 0.076 |
| FJ553090 | 0.013 | 0.013 | 0.146 | 0.013 | 0.014 | 0.003 |
| JN395308 | 0.220 | 0.221 | 0.015 | 0.102 | 0.228 | 0.011 |
| FN562027 | 0.027 | 0.156 | 0.023 | 0.002 | 0.000 | 0.002 |
| JN225904 | 0.049 | 0.006 | 0.289 | 0.004 | 0.061 | 0.084 |
| DQ249212 | 0.009 | 0.000 | 0.148 | 0.016 | 0.014 | 0.000 |
| JN396012 | 0.042 | 0.013 | 0.025 | 0.024 | 0.028 | 0.071 |
| New.ReferenceOTU6 | 0.029 | 0.102 | 0.002 | 0.001 | 0.034 | 0.038 |
| GU721975 | 0.047 | 0.040 | 0.000 | 0.005 | 0.054 | 0.077 |
| EU552140 | 0.115 | 0.009 | 0.131 | 0.019 | 0.023 | 0.029 |
| New.ReferenceOTU24 | 0.124 | 0.024 | 0.004 | 0.055 | 0.063 | 0.013 |
| New.ReferenceOTU40 | 0.274 | 0.008 | 0.025 | 0.142 | 0.160 | 0.138 |
| New.ReferenceOTU38 | 0.197 | 0.056 | 0.104 | 0.139 | 0.053 | 0.137 |
| FJ760312 | 0.332 | 0.262 | 0.035 | 0.018 | 0.230 | 0.087 |
| JQ666344 | 0.281 | 0.207 | 0.115 | 0.081 | 0.164 | 0.068 |
| AB044636 | 0.087 | 0.152 | 0.000 | 0.005 | 0.075 | 0.107 |
| New.ReferenceOTU117 | 0.021 | 0.000 | 0.032 | 0.080 | 0.088 | 0.037 |
| New.ReferenceOTU44 | 0.276 | 0.261 | 0.086 | 0.018 | 0.134 | 0.026 |
| FJ882010 | 0.107 | 0.039 | 0.010 | 0.120 | 0.104 | 0.081 |
| GU370752 | 0.129 | 0.043 | 0.009 | 0.058 | 0.114 | 0.000 |
| New.ReferenceOTU37 | 0.003 | 0.091 | 0.032 | 0.000 | 0.012 | 0.000 |
| AB303550 | 0.052 | 0.032 | 0.001 | 0.009 | 0.055 | 0.079 |
| New.ReferenceOTU42 | 0.063 | 0.044 | 0.003 | 0.001 | 0.065 | 0.048 |
| New.ReferenceOTU162 | 0.011 | 0.049 | 0.017 | 0.041 | 0.005 | 0.013 |
| JF449592 | 0.328 | 0.177 | 0.044 | 0.033 | 0.243 | 0.003 |
| New.ReferenceOTU68 | 0.019 | 0.001 | 0.007 | 0.150 | 0.040 | 0.114 |
| New.ReferenceOTU122 | 0.129 | 0.000 | 0.051 | 0.586 | 0.090 | 0.494 |
| JQ410174 | 0.069 | 0.011 | 0.093 | 0.179 | 0.054 | 0.092 |
| HQ608112 | 0.028 | 0.000 | 0.005 | 0.165 | 0.053 | 0.110 |
| GU122889 | 0.031 | 0.011 | 0.059 | 0.362 | 0.042 | 0.243 |
| New.ReferenceOTU129 | 0.036 | 0.000 | 0.006 | 0.266 | 0.056 | 0.207 |
| JN905688 | 0.332 | 0.088 | 0.013 | 0.635 | 0.242 | 0.553 |
| New.ReferenceOTU63 | 0.407 | 0.533 | 0.016 | 0.051 | 0.197 | 0.034 |
| New.ReferenceOTU149 | 0.274 | 0.361 | 0.025 | 0.027 | 0.118 | 0.024 |
| New.CleanUp.ReferenceOTU4229 | 0.001 | 0.340 | 0.006 | 0.087 | 0.101 | 0.000 |
| EU620143 | 0.357 | 0.132 | 0.283 | 0.198 | 0.100 | 0.210 |
| FJ553470 | 0.033 | 0.001 | 0.013 | 0.182 | 0.075 | 0.206 |
| GQ921751 | 0.065 | 0.004 | 0.005 | 0.289 | 0.068 | 0.228 |
| New.ReferenceOTU124 | 0.009 | 0.038 | 0.278 | 0.023 | 0.016 | 0.009 |
| New.ReferenceOTU53 | 0.024 | 0.172 | 0.023 | 0.023 | 0.302 | 0.055 |
| New.CleanUp.ReferenceOTU109 | 0.111 | 0.068 | 0.104 | 0.479 | 0.085 | 0.323 |
| New.ReferenceOTU58 | 0.043 | 0.032 | 0.011 | 0.225 | 0.054 | 0.192 |
| New.ReferenceOTU70 | 0.052 | 0.004 | 0.003 | 0.280 | 0.047 | 0.200 |
| New.ReferenceOTU92 | 0.056 | 0.000 | 0.000 | 0.208 | 0.150 | 0.086 |
| New.CleanUp.ReferenceOTU181 | 0.030 | 0.000 | 0.007 | 0.165 | 0.043 | 0.127 |
| New.CleanUp.ReferenceOTU3277 | 0.056 | 0.021 | 0.006 | 0.163 | 0.037 | 0.067 |
| GQ999249 | 0.034 | 0.024 | 0.007 | 0.100 | 0.016 | 0.113 |
| New.ReferenceOTU9 | 0.062 | 0.136 | 0.000 | 0.151 | 0.000 | 0.104 |
| New.ReferenceOTU74 | 0.030 | 0.000 | 0.012 | 0.000 | 0.244 | 0.235 |
| AB490728 | 0.089 | 0.085 | 0.096 | 0.006 | 0.188 | 0.005 |

(b)

| Zheng58 Group | OC | AK | TN | AN | TP | AP |
| --- | --- | --- | --- | --- | --- | --- |
| New.ReferenceOTU41 | 0.056 | 0.000 | 0.005 | 0.167 | 0.004 | 0.085 |
| New.ReferenceOTU79 | 0.019 | 0.002 | 0.092 | 0.011 | 0.000 | 0.011 |
| HQ832844 | 0.224 | 0.128 | 0.062 | 0.091 | 0.093 | 0.007 |
| GU721975 | 0.001 | 0.000 | 0.002 | 0.011 | 0.040 | 0.003 |
| EU489956 | 0.013 | 0.000 | 0.023 | 0.006 | 0.000 | 0.083 |
| EU490138 | 0.043 | 0.056 | 0.018 | 0.016 | 0.060 | 0.003 |
| New.ReferenceOTU142 | 0.007 | 0.145 | 0.153 | 0.002 | 0.000 | 0.008 |
| New.ReferenceOTU92 | 0.035 | 0.002 | 0.009 | 0.022 | 0.016 | 0.016 |
| New.ReferenceOTU59 | 0.132 | 0.048 | 0.000 | 0.014 | 0.045 | 0.002 |
| AB490728 | 0.174 | 0.049 | 0.030 | 0.016 | 0.120 | 0.003 |
| JN225904 | 0.011 | 0.019 | 0.011 | 0.001 | 0.138 | 0.078 |
| EU301639 | 0.107 | 0.032 | 0.020 | 0.000 | 0.203 | 0.009 |
| New.ReferenceOTU159 | 0.000 | 0.013 | 0.111 | 0.050 | 0.028 | 0.015 |
| New.ReferenceOTU74 | 0.011 | 0.068 | 0.092 | 0.004 | 0.054 | 0.075 |
| JF497127 | 0.182 | 0.031 | 0.000 | 0.064 | 0.030 | 0.182 |
| AM901894 | 0.028 | 0.000 | 0.000 | 0.019 | 0.066 | 0.004 |
| EU490021 | 0.030 | 0.031 | 0.111 | 0.044 | 0.002 | 0.000 |
| HQ608112 | 0.092 | 0.005 | 0.082 | 0.044 | 0.095 | 0.023 |
| HM069461 | 0.022 | 0.005 | 0.046 | 0.010 | 0.004 | 0.001 |
| GQ130305 | 0.058 | 0.000 | 0.050 | 0.039 | 0.268 | 0.009 |
| AY999117 | 0.031 | 0.003 | 0.285 | 0.000 | 0.025 | 0.010 |
| New.ReferenceOTU47 | 0.030 | 0.006 | 0.047 | 0.006 | 0.059 | 0.007 |
| New.ReferenceOTU45 | 0.012 | 0.027 | 0.000 | 0.044 | 0.000 | 0.114 |
| New.ReferenceOTU18 | 0.009 | 0.000 | 0.016 | 0.000 | 0.221 | 0.023 |
| HM162214 | 0.030 | 0.000 | 0.125 | 0.341 | 0.035 | 0.277 |
| JN905688 | 0.056 | 0.000 | 0.091 | 0.029 | 0.216 | 0.009 |
| AB303550 | 0.000 | 0.016 | 0.070 | 0.011 | 0.045 | 0.062 |
| New.ReferenceOTU9 | 0.071 | 0.006 | 0.002 | 0.068 | 0.071 | 0.016 |
| JN396012 | 0.205 | 0.001 | 0.214 | 0.087 | 0.074 | 0.143 |
| New.ReferenceOTU38 | 0.019 | 0.002 | 0.002 | 0.000 | 0.000 | 0.045 |
| New.ReferenceOTU0 | 0.045 | 0.038 | 0.029 | 0.151 | 0.004 | 0.243 |
| GU045770 | 0.202 | 0.020 | 0.059 | 0.041 | 0.186 | 0.005 |
| New.ReferenceOTU44 | 0.118 | 0.032 | 0.010 | 0.002 | 0.028 | 0.011 |
| FJ783707 | 0.014 | 0.014 | 0.084 | 0.014 | 0.000 | 0.001 |
| New.ReferenceOTU124 | 0.048 | 0.011 | 0.102 | 0.343 | 0.017 | 0.227 |
| FN562027 | 0.020 | 0.025 | 0.131 | 0.046 | 0.000 | 0.000 |
| New.ReferenceOTU105 | 0.023 | 0.040 | 0.203 | 0.002 | 0.069 | 0.082 |
| JF439466 | 0.006 | 0.005 | 0.044 | 0.004 | 0.061 | 0.039 |
| JN904546 | 0.013 | 0.032 | 0.115 | 0.040 | 0.000 | 0.040 |
| New.ReferenceOTU30 | 0.013 | 0.339 | 0.013 | 0.035 | 0.000 | 0.149 |
| New.ReferenceOTU133 | 0.137 | 0.085 | 0.001 | 0.043 | 0.101 | 0.003 |
| GU370752 | 0.006 | 0.091 | 0.006 | 0.154 | 0.002 | 0.073 |
| New.CleanUp.ReferenceOTU3277 | 0.003 | 0.059 | 0.130 | 0.012 | 0.005 | 0.007 |
| New.ReferenceOTU117 | 0.017 | 0.018 | 0.092 | 0.107 | 0.021 | 0.107 |
| EU620134 | 0.000 | 0.067 | 0.008 | 0.067 | 0.022 | 0.039 |
| JF449592 | 0.094 | 0.005 | 0.114 | 0.380 | 0.011 | 0.406 |
| AY123741 | 0.078 | 0.015 | 0.329 | 0.467 | 0.030 | 0.424 |
| New.ReferenceOTU68 | 0.028 | 0.004 | 0.185 | 0.369 | 0.029 | 0.435 |
| New.ReferenceOTU11 | 0.119 | 0.022 | 0.142 | 0.281 | 0.002 | 0.300 |
| New.ReferenceOTU58 | 0.117 | 0.050 | 0.008 | 0.000 | 0.263 | 0.008 |
| JQ666344 | 0.142 | 0.009 | 0.071 | 0.013 | 0.179 | 0.022 |
| New.ReferenceOTU100 | 0.096 | 0.020 | 0.137 | 0.279 | 0.005 | 0.305 |
| New.ReferenceOTU24 | 0.142 | 0.041 | 0.006 | 0.030 | 0.012 | 0.107 |
| New.ReferenceOTU122 | 0.252 | 0.003 | 0.270 | 0.663 | 0.005 | 0.429 |
| FJ783068 | 0.003 | 0.067 | 0.002 | 0.003 | 0.060 | 0.039 |
| New.ReferenceOTU40 | 0.096 | 0.026 | 0.038 | 0.172 | 0.022 | 0.076 |
| JQ410174 | 0.024 | 0.004 | 0.000 | 0.105 | 0.004 | 0.037 |
| New.ReferenceOTU162 | 0.088 | 0.008 | 0.008 | 0.129 | 0.044 | 0.118 |
| New.ReferenceOTU53 | 0.023 | 0.018 | 0.090 | 0.006 | 0.000 | 0.013 |
| New.ReferenceOTU36 | 0.276 | 0.028 | 0.302 | 0.508 | 0.018 | 0.510 |
| FJ466722 | 0.109 | 0.052 | 0.144 | 0.277 | 0.004 | 0.334 |
| New.ReferenceOTU6 | 0.156 | 0.024 | 0.120 | 0.282 | 0.010 | 0.304 |
| FJ553470 | 0.166 | 0.024 | 0.241 | 0.122 | 0.001 | 0.227 |
| EU547495 | 0.007 | 0.090 | 0.077 | 0.214 | 0.032 | 0.263 |
| JN395308 | 0.003 | 0.041 | 0.079 | 0.179 | 0.022 | 0.213 |
| New.ReferenceOTU37 | 0.094 | 0.021 | 0.142 | 0.278 | 0.006 | 0.308 |
| New.CleanUp.ReferenceOTU181 | 0.145 | 0.035 | 0.243 | 0.606 | 0.011 | 0.745 |
| New.ReferenceOTU129 | 0.175 | 0.023 | 0.195 | 0.674 | 0.016 | 0.691 |
| New.CleanUp.ReferenceOTU4229 | 0.179 | 0.112 | 0.153 | 0.044 | 0.111 | 0.011 |
| New.ReferenceOTU42 | 0.098 | 0.009 | 0.189 | 0.377 | 0.011 | 0.398 |
| FN610923 | 0.105 | 0.017 | 0.147 | 0.284 | 0.007 | 0.304 |
| New.CleanUp.ReferenceOTU4682 | 0.002 | 0.277 | 0.175 | 0.011 | 0.020 | 0.027 |
| New.CleanUp.ReferenceOTU1969 | 0.065 | 0.170 | 0.288 | 0.061 | 0.015 | 0.015 |
| FN812833 | 0.141 | 0.029 | 0.210 | 0.674 | 0.004 | 0.573 |
| New.ReferenceOTU154 | 0.217 | 0.066 | 0.063 | 0.257 | 0.021 | 0.210 |
| New.ReferenceOTU108 | 0.218 | 0.125 | 0.292 | 0.359 | 0.007 | 0.449 |
| JF497141 | 0.022 | 0.110 | 0.002 | 0.000 | 0.121 | 0.015 |
| New.ReferenceOTU149 | 0.019 | 0.004 | 0.190 | 0.041 | 0.002 | 0.046 |
| FJ553090 | 0.001 | 0.019 | 0.016 | 0.026 | 0.067 | 0.042 |
| EU552140 | 0.145 | 0.103 | 0.164 | 0.273 | 0.002 | 0.330 |
| FJ760312 | 0.049 | 0.030 | 0.031 | 0.056 | 0.019 | 0.057 |
| New.CleanUp.ReferenceOTU3978 | 0.113 | 0.083 | 0.001 | 0.027 | 0.143 | 0.000 |
| FJ760641 | 0.041 | 0.021 | 0.049 | 0.032 | 0.000 | 0.019 |
| New.CleanUp.ReferenceOTU801 | 0.007 | 0.110 | 0.034 | 0.007 | 0.015 | 0.020 |
| FJ882010 | 0.406 | 0.175 | 0.001 | 0.296 | 0.355 | 0.082 |
| GQ921751 | 0.046 | 0.127 | 0.019 | 0.247 | 0.025 | 0.045 |
| New.CleanUp.ReferenceOTU4674 | 0.210 | 0.005 | 0.360 | 0.425 | 0.011 | 0.351 |
